# New Scenarios of Chagas Disease Transmission in Northern Colombia

**DOI:** 10.1155/2017/3943215

**Published:** 2017-09-26

**Authors:** Catalina Tovar Acero, Jorge Negrete Peñata, Camila González, Cielo León, Mario Ortiz, Julio Chacón Pacheco, Elkin Monterrosa, Abraham Luna, Dina Ricardo Caldera, Lyda Espitia-Pérez

**Affiliations:** ^1^Grupo de Investigación en Enfermedades Tropicales y Resistencia Bacteriana, Facultad de Ciencias de la Salud, Universidad del Sinú, Montería, Colombia; ^2^Laboratorio de Investigaciones Biomédicas, Universidad del Sinú, Montería, Colombia; ^3^Departamento de Ciencias Biológicas, Centro de Investigaciones en Microbiología y Parasitología Tropical (CIMPAT), Universidad de los Andes, Bogotá, Colombia; ^4^Fundación Colombia Mia, Montería, Colombia; ^5^Grupo de Investigación Biodiversidad Unicordoba, Universidad de Córdoba, Montería, Colombia; ^6^Área de Entomología, Laboratorio de Salud Pública de Córdoba, Montería, Colombia; ^7^Hospital San Juan de Sahagún, Sahagún, Colombia; ^8^Grupo de Investigación Biomédica y Biología Molecular, Facultad de Ciencias de la Salud, Universidad del Sinú, Montería, Colombia

## Abstract

Chagas disease (CD) is a systemic parasitic infection caused by the flagellated form of* Trypanosoma cruzi*. Córdoba department, located in the Colombian Caribbean Coast, was not considered as a region at risk of* T. cruzi *transmission. In this article, we describe the first acute CD case in Salitral village in Sahagún, Córdoba, confirmed by microscopy and serological tests. Our results draw attention to a new scenario of transmission of acute CD in nonendemic areas of Colombia and highlight the need to include CD in the differential diagnosis of febrile syndromes in this region.

Chagas disease (CD) also known as American Trypanosomiasis is a systemic parasitic infection caused by the protozoan parasite* Trypanosoma cruzi (T. cruzi),* which affects six to seven million people worldwide with an annual incidence of 28.000 cases in the Americas [1]. Transmission to humans as well as to domestic and sylvatic mammals occurs mainly through the introduction of the parasite present in triatomine bug feces during its blood meals; however, alternate transmission routes include blood transfusion, organ transplants, laboratory accidents, congenital and oral ingestion of contaminated food [2, 3]. During the acute phase of the disease up to 30% of patients suffer from cardiac disorders and up to 10% suffer from digestive (typical enlargement of the esophagus, spleen, or colon), neurological, or mixed alterations [4]. Colombia has been one of the Latin American countries with a considerable number of acute Chagas disease outbreaks where oral transmission of* T. cruzi* has been recorded specially in endemic areas of Santander, Norte de Santander, Cundinamarca, Boyacá, Casanare, Meta, Arauca, and some areas of the Sierra Nevada of Santa Marta [5]. Córdoba department located in the Colombian Caribbean Coast is not considered as an endemic region for* T. cruzi *transmission; therefore CD is not included in the diagnosis of febrile diseases in hospitals and health centres. Additionally, due to the lack of knowledge about CD clinical symptoms, diagnosis in a setting where multiple infectious tropical diseases are present, such as tuberculosis, malaria, dengue, chikungunya, and Zika, is a challenge; therefore annual reports considered CD cases to be imported from other departments. In this article, we describe the first acute CD case in Salitral village in Sahagún, Córdoba, confirmed by microscopy and serological tests. Sampling area was located in Salitral village in the Sahagún municipality of Córdoba department, located in the northwest part of Colombia (8°49′47.9′′N, 75°31′31.5′′W, and 75 m.a.s.l.) (Figure 1). This area has a tropical climate with an annual mean temperature between 27°C and 30°C and a relative humidity of 84%. Main economic activities are related to mixed extensive crop-livestock systems, usually linked to the proliferation of wild small mammals (rodents and marsupials) described as* T. cruzi *reservoirs.

The 16-year-old young male patient born and resident in Salitral village was referred to the emergency service of San Juan de Sahagún Hospital (ESE HSJS) with eight days of headache and high fever (38°C) associated with chills, generalized myalgia, asthenia, adynamia, choluria, severe epigastralgia without epistaxis, and gingivorrhagia. On his epidemiological history, the patient denied knowing triatomine bugs, having received any blood transfusion or organ transplant, or traveling outside Córdoba prior to the beginning of the symptoms. No inoculation point either in skin or periocular region suggesting vectorial transmission could be detected. The patient was alert, without respiratory distress or cardiovascular involvement (electrocardiogram). Abdominal palpation and ultrasound examination confirmed symptoms of a moderate spleen enlargement (splenomegaly). Thick blood smear examination was negative for* Plasmodium* spp. but positive for* T. cruzi *trypomastigotes (Figure 2). Serological analysis by enzyme linked immunosorbent assay (ELISA) for CD was negative at the seventh day of hospitalization.

All experimental and sampling protocols were approved by the Ethics Committee of Universidad del Sinú according to national normativity for human populations studies and the NIH Guide for the Care and Use of Animals [6].

In order to determine the presence of triatomine bugs, active manual search was carried out by the professional staff and community members in walls, cracks in the walls and ceiling, mattresses, and floor of the patient's house and other 24 houses in the neighborhood area according to OMS recommended methodology [7]. Additionally, live-baited traps [8] and Gómez-Nuñez boxes [9] were also placed in the intra and peridomicile of each selected household. Taxonomic identification of captured specimens was performed based on external morphology, according to Lent and Wygodzinsky [10]. Detection of* T. cruzi *infection in captured triatomines was confirmed by direct and molecular techniques examining intestinal contents and rectal ampulla. Small- and mid-sized mammals were also captured using 5 mist nets for bats and 20 Tomahawks and 40 Sherman traps. Captured mammals were taxonomically identified according to Emmons and Feer [11], Linares [12], Tirira [13], Gardner [14], and Patton et al. [15] and whole blood samples were taken for molecular identification of* T. cruzi. *Sampling was performed on 80 volunteers selected from the entire population. All participants filled out a clinical-epidemiological survey including identification variables and evidence of signs or symptoms according to case definition. All family members and other individuals related to the acute case patient were also analyzed. All samples were collected after obtaining the corresponding informed consent. The serological analysis included detection of IgG antibodies by ELISA and indirect immunofluorescence (IFI). For* T. cruzi* detection, human blood samples and triatomine bugs rectal ampulla were collected in a volume solution containing EDTA and guanidine 6M and stored at room temperature. A spin column-based nucleic acid purification kit was used to perform DNA extraction (High Pure PCR Template Preparation, de Roche®). Molecular detection was carried out through amplification of the variable region of kinetoplast DNA (kDNA) according to the methodology previously described by [16, 17] and tandem repeat satellite region from* T. cruzi* using the* cruzi1 *and* cruzi2* primers described by [18]. Amplification cycles for kDNA were performed using a two-step procedure using an initial denaturation step at 94°C for 3 min; 5 cycles of denaturation at 94°C for 1 min, annealing at 68°C for 1 min, and extension at 72°C for 1 min, followed by 35 cycles at 94°C for 45 sec; annealing at 64°C for 45 sec, extension at 72°C for 45 sec, and final extension at 72°C for 10 min. Cycling conditions for* cruzi1* and* cruzi2 *were initial denaturation at 94°C for 5 min and 40 cycles of denaturation at 94°C for 1 min, annealing at 64°C for 30 sec, extension at 72°C for 1 min, and final extension at 72°C for 10 min. Patient's house was built with wood walls, palm roofs, and dirt floors surrounded by dense vegetation consisting of trees and palms (Figure 3). Among the 24 houses included in the study 32% were constructed with wooden walls, 40% with dirt floors, and 56% with thatch palm roof and 32% had unplastered walls. Conventional parasitological methods, serological screening, and molecular testing for detection of* T. cruzi* infection performed on blood samples of 80 voluntary patients showed negative results. In this particular community, serological test showed positive results only for the acute case described in this work. During the entomological sampling, seven individuals identified as* Rhodnius pallescens* and two classified as* Panstrongylus geniculatus* were captured. Most captured insects were collected by members of the community. Analysis of intestinal content and rectal ampulla confirmed the presence of* T. cruzi *in one specimen of each species. Analysis of blood samples of 29 specimens of small- and mid-sized mammals using molecular methods confirmed the presence of* T. cruzi* DNA in two specimens of* Didelphis marsupialis* and two specimens of* Heteromys anomalus*. The transmission scenario of CD in Córdoba still remains a challenge and must be addressed through clinical and ecoepidemiological studies since, as our results showed, a sylvatic cycle exists and accidental human cases might be occurring. In this particular case, signs and symptoms presented by the patient including prolonged febrile illness, epigastralgia, and absence of lesions in either the skin or the periocular region indicating the insect bite, together with patient statement of never being bitten by triatomines and never leaving Córdoba department, would suggest oral transmission as the most likely pathway of infection [19, 20].

Considering the* T. cruzi *detection in specimens of the triatomine bugs* Panstrongylus geniculatus *and* Rhodnius pallescens *and the mammals* Didelphis marsupialis* and* Heteromys anomalus, *there is an evident risk of infection to humans either. In Córdoba department previous studies reported the presence of several triatomine species [21]. In line with our findings,* E. cuspidatus, P. geniculatus, *and* R. pallescens* had previously been reported in Sahagún municipality [22]. However, no evidence of domiciliated triatomines was found. Even when no evidence of domiciliated* P. geniculatus* has been reported for Colombia, recent reports about the increasing frequency of* Panstrongylus* species displaying ability to invade and colonize human habitats are focusing the interest of entomologists and CD control managers throughout Latin America [23]. Current land use changes in Colombia and particularly in Córdoba, where vast forested areas have been cleared for livestock and agricultural activities, may favor triatomine domiciliation. This new situation could impose necessary changes in the strategy of CD control programs in Colombia, which until now have been limited to vector control activities in rural communities in endemic areas. Community engagement in sampling activities constituted a very effective approach for triatomine collection [24]. In our case, triatomine collection by community members was 3.5 times more effective compared to conventional sampling. These data are of key importance for the successful implementation of vector control in Córdoba and community participation could be a method of choice for sustained monitoring of triatomines in this area. Considering that there were no previous reports of* T. cruzi* infected reservoirs in Córdoba, our study represents the first report of* T. cruzi* infection detected in small mammals (*Didelphis marsupialis* and* Heteromys anomalus*) from this particular region. As documented by Cantillo-Barraza et al. [25],* D. marsupialis* may play an active role in the amplification of* T. cruzi* transmission in peridomestic areas mediating enzootic cycle or acting as a link between the enzootic and domestic cycles. In previous studies,* Heteromys anomalus* has been associated with sylvatic transmission cycles of CD in Colombia [26]. Our study also confirmed* T. cruzi* transmission in Salitral municipality evidenced by the presence of the parasite in different actors involved in the transmission cycle (human, reservoir, and vector). Even when no domiciliated vectors were found, our findings suggest the existence of autochthonous human cases in Córdoba and highlight the need to include CD in the differential diagnosis of febrile syndromes and diseases of this region. Despite the improvements in building materials and construction conditions of human dwellings in Córdoba, in some rural areas contact with natural and sylvatic environments persists, thereby creating the constant presence of potential vectors and reservoirs like triatomines, marsupials, and small mammals around the peridomicile. This close contact presumably could enable the emergence of CD cases [27]. Similarly, in rural areas some practices related to food preparation and storage may also constitute a potential risk factor increasing the contact with triatomine feces and small mammals dejections [28]. This study draws attention to new scenarios transmission of CD in nonendemic areas of Córdoba department in Colombia and highlights the need to include CD in the differential diagnosis of febrile syndromes and diseases of this region.

## Figures and Tables

**Figure 1:**
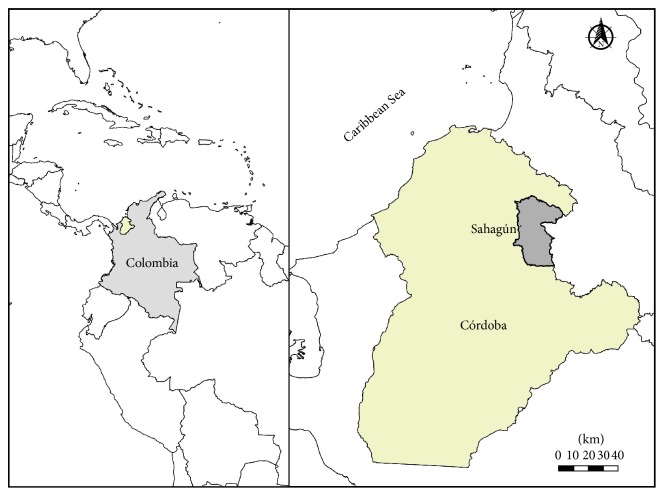
Sampling area (Salitral village, Córdoba, Colombia).

**Figure 2:**
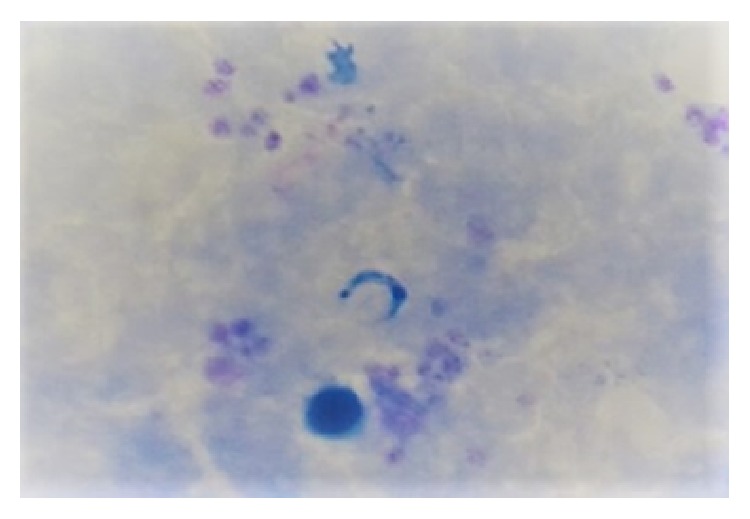
*T. cruzi *trypomastigotes detected in thick blood smears of the infected patient (1000x).

**Figure 3:**
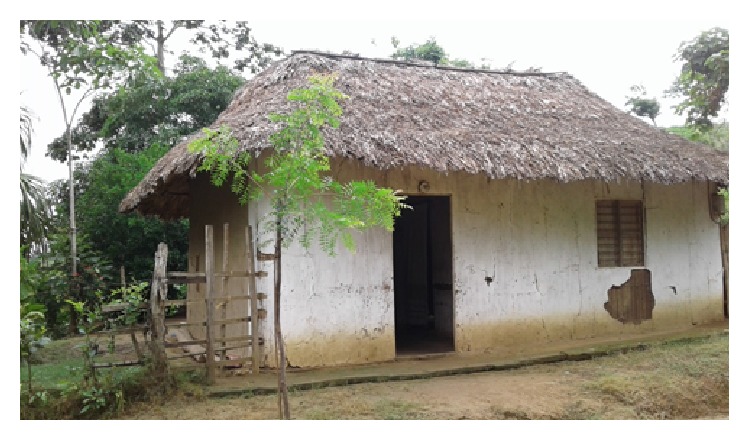
Patient's house infrastructure and peridomicile.
